# Interaction with Pyruvate Kinase M2 Destabilizes Tristetraprolin by Proteasome Degradation and Regulates Cell Proliferation in Breast Cancer

**DOI:** 10.1038/srep22449

**Published:** 2016-03-01

**Authors:** Liangqian Huang, Zhenhai Yu, Zhenchao Zhang, Wenjing Ma, Shaoli Song, Gang Huang

**Affiliations:** 1 Institute of Health Sciences, Shanghai Institutes for Biological Sciences (SIBS), Chinese Academy of Sciences (CAS) & Shanghai Jiao Tong University School of Medicine (SJTUSM), Shanghai 200025, China; 2 Department of Nuclear Medicine, Renji Hospital, School of Medicine, Shanghai Jiao Tong University, Shanghai 200127, China; 3 School of Biomedical Engineering, Shanghai Jiao Tong University, Shanghai 200030, China

## Abstract

Pyruvate kinase M2 (PKM2), which is predominantly expressed in most cancers, plays a key role in the Warburg effect. However, how PKM2 functions as a tumor supportive protein has not been fully elucidated. Here, we identified tristetraprolin (TTP), an AU-rich, element-binding protein that regulates mRNA stability, as a new binding partner of PKM2. Our data reveal that PKM2 suppresses TTP protein levels by promoting its phosphorylation, ubiquitination, and proteasome degradation, reducing its mRNA turnover ability and ultimately impairing cell viability in breast cancer cells. The p38/mitogen-activated protein kinase (MAPK) pathway might be involved in PKM2-mediated TTP degradation, while treatment with the p38 inhibitor or siRNA abolished PKM2-induced TTP protein degradation. These findings demonstrate that PKM2–TTP association is crucial for regulating breast cancer cell proliferation and is therefore a potential therapeutic target in cancer.

Compared to normal tissue, most tumors have significantly increased glucose utilization. In cancer cells, there are increased glucose consumption rates and high lactate production in the presence of oxygen, which is known as aerobic glycolysis (the Warburg effect)^1^,^2^, of which pyruvate kinase (PK) is considered a key regulator. PK is a key rate-limiting enzyme that catalyzes the final step of glycolysis, converting phosphoenolpyruvate to pyruvate while phosphorylating adenosine diphosphate (ADP) to adenosine triphosphate (ATP). There are four PK isoforms encoded by two separate genes: PKL, PLR, PKM1, and PKM2. PKL and PKR originate from the *PKL* gene by alternative splicing, and they are expressed tissue-specifically in the liver and red blood cells, respectively^3^. PKM1 and PKM2 are alternative splicing products of the *PKM* gene (exon 9, PKM1; exon 10, PKM2). During tumorigenesis, PKM1/L/R expression gradually diminishes, and PKM2 expression replaces it, suggesting the unique role of PKM2 in cancer cells^4^. As PKM2 enzymatic activity is much lower than that of PKM1, it channels more glycolytic intermediates, e.g., nucleic acids, amino acids, and lipids, into building blocks, further supporting cancer cell proliferation^2^. In addition to its direct roles in glycolysis, recent studies have demonstrated that PKM2 can function as a transcriptional co-activator or protein kinase to promote tumorigenesis^5^,^6^. It can phosphorylate histone H3, signal transducer and activator of transcription 3 (STAT3), or myosin light chain 2 (MLC2) to activate transcription, and interacts with other proteins, such as β-catenin, Oct-4, and HIF-1α, to exert its function as a transcription co-factor^2^,^7^,^8^. PKM2 also interacts with CD44, enhancing the glycolytic phenotype of cancer cells. Recent research shows that PKM2 interacts with P65 and the PKM2/NF-κB/microRNA (miR)-148a/152 feedback loop, which regulates cancer cell growth and angiogenesis in response to insulin-like growth factor 1 receptor (IGF-IR) activation in breast cancer cells^9^. However, the molecular mechanisms underlying PKM2 function as an tumor supportive protein require further clarification.

The tandem zinc finger protein tristetraprolin (TTP), also known as Nup475, Tis11, or Zfp36, is an AU-rich element (ARE)-binding protein that belongs to the *TIS11*/*TTP* gene family, regulating the stability of multiple target mRNAs^10^. In addition to its function in immune response, TTP is also involved in cell differentiation, apoptosis, and tumorigenesis^11^. TTP binds and destabilizes the mRNAs encoding cytokines and proto-oncogenes such as c-MYC, tumor necrosis factor α (TNFα), granulocyte monocyte colony stimulating factor (GM-CSF), interleukin-2 (IL2), cyclooxygenase 2 (COX-2), vascular endothelial growth factor (VEGF), nuclear factor κB (NF-κB), and hypoxia-inducible factor 1a (HIF-1a), which has a significant effect on cell viability, indicating a possible role for TTP in angiogenesis and tumor growth^12^,^13^,^14^,^15^,^16^. TTP may also regulate its own expression by binding to an ARE in the 3′ untranslated region of *TTP* mRNA^17^. Recent studies suggest that TTP has tumor suppressor activities. It is down-regulated or hypermodified and therefore inactive in many cancer cells, including that of thyroid, lung, ovary, uterus, and breast cancer, as compared with non-transformed cell types^11^,^18^. Kinases such as protein kinase B (PKB)/AKT, p38 MAPK, MK2, extracellular signal–regulated kinase 1 (ERK1), MEKK1, and c-Jun N-terminal kinase (JNK) can phosphorylate TTP^17^,^19^,^20^,^21^. Among these protein kinases, the p38 MAPK/MK2 pathway is a crucial regulator of TTP^22^. TTP protein is unstable and is rapidly degraded by proteasomes; however, TTP phosphorylation by p38 MAPK protects it from proteasome degradation and disables its mRNA turnover ability. Johnson and colleagues found that TTP phosphorylation by MK2 increases 14-3-3 protein binding^23^. The 14-3-3 proteins bind specifically to the TTP C-terminal region sequence, thereby excluding TTP from stress granules, inactivating TTP and protecting it from proteasome proteolysis *in vivo*^24^,^25^. Despite these studies on TTP modification, little is known of the molecular mechanism responsible for the manipulation of TTP stability. Breast cancer is the most common malignant cancer among women, comprising almost a fifth of all female cancers, and with high incidence and mortality rates^26^. Previous works have demonstrated that TTP expression is comparatively lower in invasive breast cancer cells as compared with the normal breast cell lines^27^. Thus, knowledge of TTP induction and suppression may lead to new insights into the role of TTP deficiency in breast cancer processes.

In this study, PKM2 was identified as a binding partner of TTP both *in vitro* and *in vivo*. Our data demonstrated that PKM2 bound to the TTP N-terminus and promoted TTP phosphorylation, ubiquitination, and proteasome degradation. Inhibiting p38 mitogen-activated protein kinase (MAPK) by siRNA or inhibitor SB203580 prevented TTP degradation, which indicated that the p38 pathway might be involved in PKM2-mediated TTP degradation. Moreover, manipulating PKM2 protein levels altered mRNA degradation activity of TTP. Our data also revealed that the association between PKM2 and TTP was important in breast cancer during cell proliferation. Our findings provide new insight into the mechanism underlying TTP regulation by PKM2, which may be a potential therapy target in breast cancer.

## Results

### PKM2 interacts with TTP *in vitro* and *in vivo*

**Figure 1:**
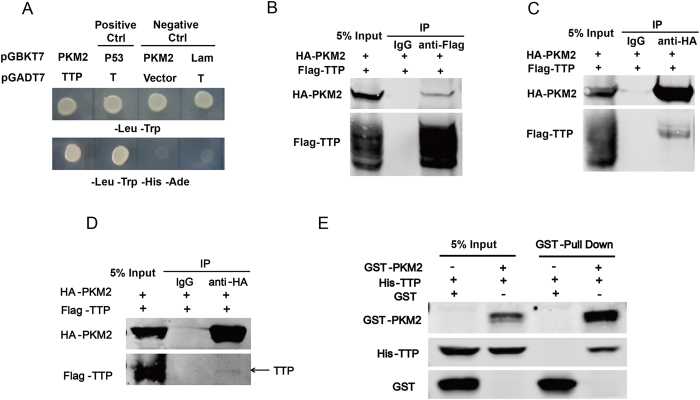
PKM2 interacts with TTP *in vitro* and *in vivo*. (**A**) Interaction between PKM2 and TTP was re-examined by yeast two-hybrid using nutritional deficient culture medium (SD-Leu-Trp or SD-Leu-Trp-His-Ade). Vector pGADT7-T with pGBKT7-p53 or pGBKT7-Lam was used as positive control or negative control, respectively. (**B,C**) Interaction between Flag-tagged TTP and HA-tagged PKM2 full-length proteins was examined by Co-IP followed by western blotting using anti-HA antibody or anti-Flag antibody. Tagged proteins were over-expressed in HEK293T cells by transient transfection. (**D**) Interaction between Flag-tagged TTP and HA-tagged PKM2 performed in MCF7 cells. (**E**) GST pull-down assays were performed to examine the direct interaction between PKM2 and TTP using recombinant His-tagged TTP and GST-tagged PKM2 purified from BL21(DE3). Gels have been run under the same experimental conditions, full-length gels are presented in Supplementary Fig. S2.

We used a yeast two-hybrid assay to screen PKM2-interacting proteins to identify proteins that may be involved in PKM2 function in cancers. The full-length N-terminal PKM2 fragment (1–354 amino acids [aa]) and two C-terminal fragments (354–531 aa and 406–531 aa) were used as bait to screen a human kidney cDNA prey library. We extracted and sequenced the positive clones baited from the library^28^. Among the candidates, TTP, an RNA-binding protein that regulates the stability of certain ARE mRNAs, was identified as a potential novel PKM2 binding partner. We re-examined the interaction in the yeast system and found that both TTP prey vector and PKM2 bait vector are required for yeast growth in nutrient-deficient SD medium (Fig. 1A).

To determine whether TTP interacted with PKM2 in mammalian cells, recombined PKM2 and TTP were generated to perform the coimmunoprecipitation experiments, which revealed that they interacted in mammalian cells (Fig. 1B–D). More importantly, glutathione S-transferase (GST) pull-down assay confirmed direct PKM2/TTP binding *in vitro* (Fig. 1E).

### PKM2 interacts with TTP protein N-terminus

**Figure 2:**
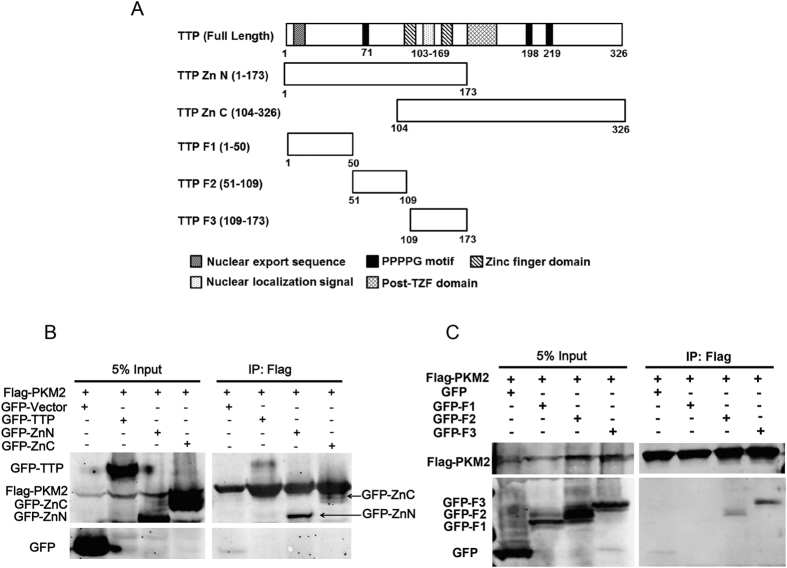
PKM2 interacts with TTP protein N-terminus. (**A**) Schematic representation of TTP protein fragments used in the mapping analysis. (**B,C**) Flag-tagged PKM2 was co-expressed in HEK293 cells with GFP-tagged TTP fragments as indicated. Co-IP followed western blotting was performed to determine the interactions. The gels have been run under the same experimental conditions, full-length gels are presented in Supplementary Fig. S2.

TTP consists of two conserved (CCCH) zinc fingers with RNA-binding properties, along with similarly sized but divergent N- and C-terminal regions^13^. To map the TTP protein putative binding region, we generated two TTP fragments: N-terminal truncation ZnN (1–173 aa) and C-terminal truncation ZnC (103–326 aa), each containing zinc fingers and an N- or C-terminus, respectively (Fig. 2A). The two fragments were fused in-frame to green fluorescent protein (GFP) to increase their size to facilitate expression and detection. The proteins were co-expressed in HEK293T cells with Flag-tagged PKM2, and then protein associations were detected by immunoprecipitation followed by immunoblotting. Figure 2B showed that the PKM2 protein interacted with the N-terminus of the TTP protein strongly and C-terminus weakly. To narrow down the binding region, we fragmented the ZnN further: GFP-F1 (1–50 aa), GFP-F2 (51–103 aa), GFP-F3 (104–173 aa). Coimmunoprecipitation showed that two TTP fragments, GFP-F2 and GFP-F3, interacted with PKM2 (Fig. 2C). Together, these data show that PKM2 binds to TTP at its 51–173 aa region, which contains the zinc finger motif of TTP.

### PKM2 decreases TTP protein levels

**Figure 3:**
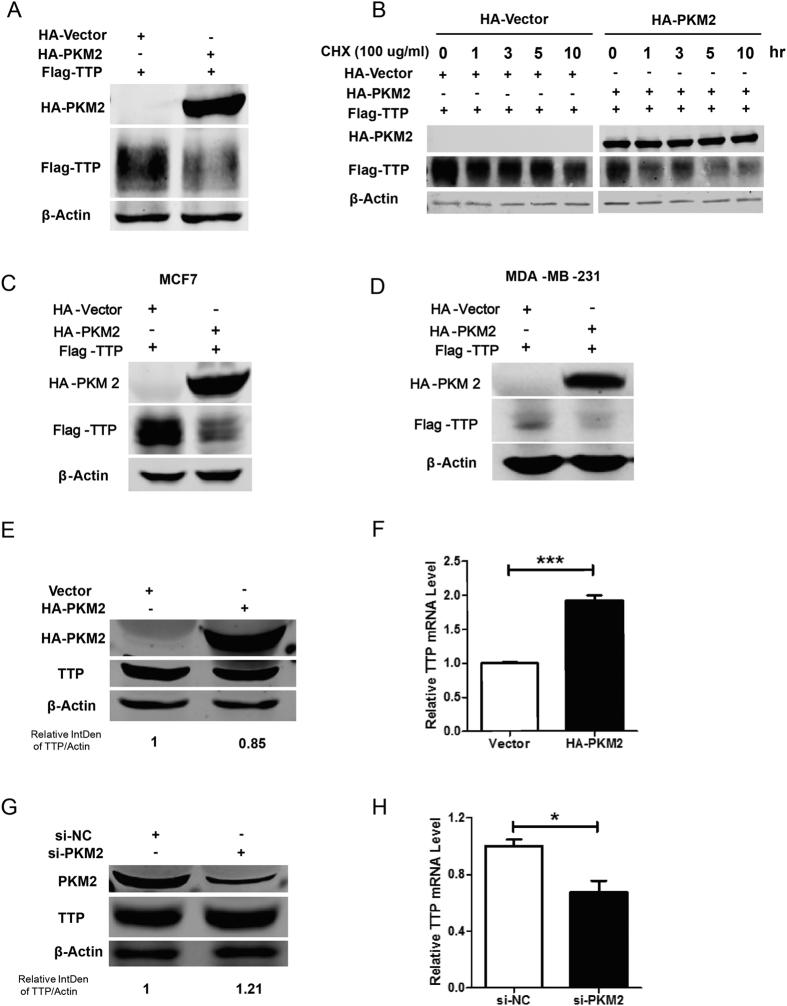
PKM2 decreases TTP protein levels. (**A**) HEK293T cells were co-transfected Flag-tagged TTP with empty vector or HA-tagged PKM2. 48 h after transfection, the protein levels were analyzed by western blotting using indicated antibody. (**B**) Flag-tagged TTP was co-transfected with empty vector or HA-tagged PKM2 in HEK293T cells. 36 h after transfection, cells were treated with 100 ug/ml CHX for indicated time, then harvested to detect TTP protein level by anti-Flag antibody. (**C,D**) Flag-tagged TTP was co-transfected with empty vector or HA-tagged PKM2 in MCF7 and MDA-MB-231 cells. 48 h after transfection, the protein levels were analyzed by western blotting using indicated antibody. (**E,F**) HA-tagged PKM2 was transfected and after 48 h incubation, endogenous TTP protein level were detected by anti-TTP antibody and mRNA level were detected by qRT-PCR. Samples derived from the same experiment and gels were processed in parallel, full-length gels are presented in Supplementary Fig. S2. (**G,H**) Endogenous PKM2 was knockdown by specific siRNA, then endogenous TTP protein level were detected by anti-TTP antibody and mRNA level were detected by qRT-PCR. (Data represent mean ± SEM n = 3).

TTP is known as a tumor suppressor^29^ while PKM2 has tumor supportive property, so we were curious about how TTP and PKM2 affected each other. In order to exam whether PKM2 affected the stability of the TTP protein, we overexpressed HA-tagged PKM2 or vector control in Flag-tagged TTP-overexpressing cells, and then detected exogenous TTP protein, which was decreased dramatically both in HEK293T (Fig. 3A) and breast cancer cell lines MCF7 and MDA-MB-231 (Fig. 3C,D). However, TTP did not impair PKM2 protein levels in the same manner. We used a cycloheximide (CHX)-based protein chase experiment to confirm the results. Figure 3B shows that, in the presence of PKM2, the half-life of TTP was reduced dramatically as compared to the control group. We also detected endogenous TTP protein and mRNA levels when manipulating PKM2 expression. PKM2 overexpression decreased endogenous TTP protein levels (Fig. 3E); conversely, *TTP* mRNA levels were increased (Fig. 3F). On the contrary, small interfering RNA (siRNA) knockdown of PKM2 increased endogenous TTP protein levels (Fig. 3G) and decreased *TTP* mRNA levels (Fig. 3H). These data demonstrate that PKM2 decreased TTP protein stability.

The changes of the endogenous TTP protein levels were not as exaggerated as that of exogenous TTP, we believe this maybe because endogenous TTP is highly modified in tumor cells, therefore, manipulating PKM2 expression only regulates TTP protein levels somewhat.

Interestingly, the changes of *TTP* mRNA levels were opposite to that of TTP protein. As previous studies have shown that *TTP* mRNA is a separate target of its own, we hypothesized that the PKM2 protein did not affect *TTP* mRNA directly, but it decreased TTP protein level, and then triggered TTP negative feedback regulation so that *TTP* mRNA eventually increased as a result.

### PKM2 enhances TTP protein phosphorylation, ubiquitination, and proteasomal degradation

**Figure 4:**
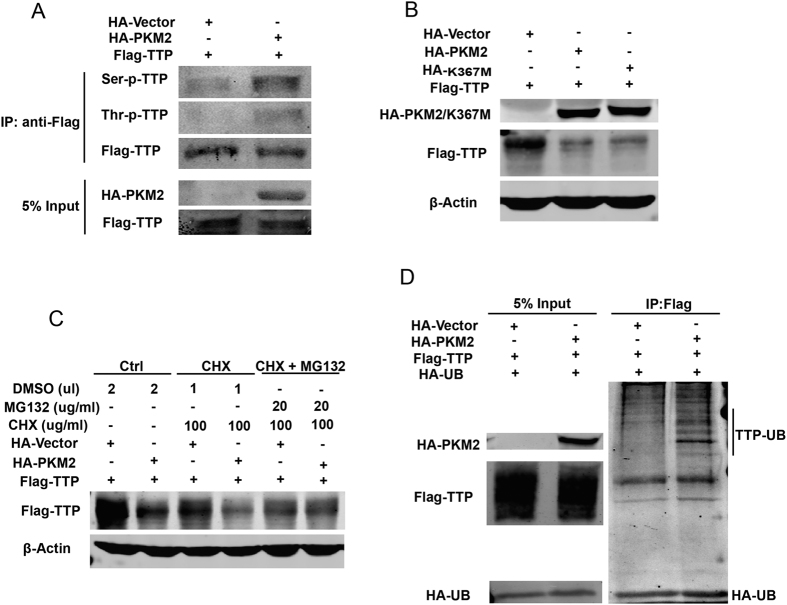
PKM2 enhances TTP protein phosphorylation, ubiquitination, and proteasomal degradation. (**A**) Flag-tagged TTP was co-transfected with empty vector or HA-tagged PKM2 in HEK293T cells. 48 h after transfection, TTP proteins were immunoprecipitated using anti-Flag antibody, and Flag-tagged TTP protein levels were normalized before phosphor-threonine or phosphor-serine levels were detected by Western blotting using indicated antibodies. (**B**) Flag-tagged TTP was co-transfected with empty vector, HA-tagged wild type PKM2 or kinase activity deficient mutant PKM2 (K367M) in HEK293T cells, then western blotting was performed to detect the TTP protein level by anti-Flag antibody. (**C**) Flag-tagged TTP was co-transfected with empty vector or HA-tagged PKM2 in HEK293T cells, 36 h after transfection cells were treated with CHX or MG132 or both, then harvested to detect by western blotting after 12 h incubation. (**D**) Cell lysates of HEK293T cells transfected with HA-tagged Ubiquitin and Flag-tagged TTP in the presence or absence of HA-tagged PKM2 were immunoprecipitated with anti-Flag antibody, the bounded proteins were detected by anti-HA antibody. Samples derived from the same experiment and gels were processed in parallel. The gels have been run under the same experimental conditions, and then were cut to incubate in different antibodies, full-length gels are presented in Supplementary Fig. S2.

To determine whether PKM2 phosphorylated TTP, HEK293T cells were transfected with Flag-tagged TTP in the presence or absence of HA-tagged PKM2. 48 h after transfection, cells were harvested to examine their phosphorylation states of exogenous TTP. Interestingly, there were increased phosphorylation levels at both serine and threonine sites (Fig. 4A), suggesting that PKM2 promotes TTP phosphorylation. To confirm whether PKM2 phosphorylated TTP itself, we generated a PKM2 protein kinase–deficient mutant PKM2(K367M)^5^,^7^,^30^. Overexpression of PKM2(K367M) degraded TTP to the same degree as the wild-type (WT) PKM2 (Fig. 4B), suggesting that although PKM2 enhances TTP protein phosphorylation, its protein kinase activity might not be involved in PKM2-mediated TTP degradation.

As previously reported, the ubiquitin–proteasome pathway is involved in TTP protein degradation^19^,^31^. To determine whether the proteasome pathway is also involved in this conditions, HEK293T cells were transfected with Flag-tagged TTP in the presence or absence of HA-tagged PKM2; 36 h after transfection, cells were treated with CHX or both CHX and the specific proteasome inhibitor MG132. 12 h post-treatment, TTP protein expression levels were examined by immunoblotting. As shown in Fig. 4C, MG132 blocked the PKM2-mediated degradation of TTP protein when protein synthesis was blocked. These findings suggest that PKM2-mediated degradation of TTP protein is dependent on the proteasome pathway.

We used an *in vivo* ubiquitination assay to assess whether TTP protein was ubiquitinated prior to its PKM2-mediated degradation. Flag-tagged TTP and HA-tagged ubiquitin were cotransfected into HEK293T cells in the presence or absence of PKM2 protein; 36 h post-transfection, cells were treated with MG132 and incubated for another 12 h. TTP proteins were then immunoprecipitated with anti-Flag antibody. As shown in Fig. 4D, there were a few protein bands of higher molecular mass detected, suggesting that TTP protein was ubiquitinated before degradation. Together, our data indicate that PKM2 increases TTP phosphorylation and ubiquitination, resulting in the promotion of proteasome degradation.

### The p38 MAPK signaling pathway is involved in PKM2-mediated TTP degradation

Various protein kinases can phosphorylate TTP. In this study, we only focused on the sites located at 51–173 aa fragment, which could be ubiquitinated or involved in TTP degradation^32^. Holper-Schichl and colleagues found that MAP 3-kinase (MEKK1)-mediated phosphorylation serves as a prerequisite for TTP lysine residue Lys63-linked polyubiquitination by TNF-associated factor 2 (TRAF2); this regulatory function depends on TTP Lys105^19^. Lee and colleagues showed that the p38 MAPK/MAPK-associated protein kinase 2 (MK2) signaling pathway could induce TTP phosphorylation, which leads to TTP protein ubiquitination and proteasomal degradation in mice^31^. Human TTP is phosphorylated by MK2 at serine residue Ser60 and Ser186^24^,^33^. We tested whether the Lys105 and Ser60 sites on the TTP protein N-terminal function in PKM2-mediated degradation of TTP.

**Figure 5:**
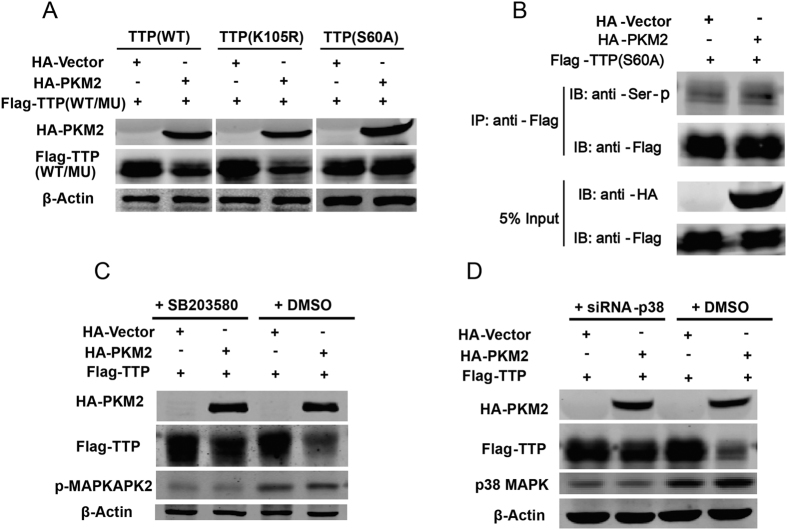
The p38 MAPK signaling pathway is involved in PKM2-mediated TTP degradation. (**A**) Flag-tagged TTP wild type (WT), mutant K105R and S60A was transfected into HEK293T cells separately in the presence or absence of HA-tagged PKM2 protein, 48 h after transfection, cells were harvested to test the TTP protein level. (**C**) Flag-tagged TTP was transfected into HEK293T cells in the presence or absence of PKM2 protein, 48 h after transfection, cells were treated with p38 inhibitor SB203580 (30 uM) and incubated for an additional 12 h, then cells were harvested to detect the protein TTP protein level. (**D**) Flag-tagged TTP was transfected into HEK293T cells in the presence or absence of PKM2 protein under the manipulation of siRNA-p38, cells were harvested to detect the protein TTP protein level 60 h after transfection. Samples derived from the same experiment and that gels were processed in parallel. The gels have been run under the same experimental conditions, and then were cut to incubate in different antibodies, full-length gels are presented in Supplementary Fig. S2.

We generated two TTP mutants: Flag-tagged TTP(K105R) and TTP(S60A). The vectors TTP WT, K105R, and S60A were transfected into HEK293T cells separately in the presence or absence of PKM2 protein; 24 h post-transfection, cells were harvested to test the TTP protein level. As shown in Fig. 5A, PKM2 protein overexpression degraded the TTP mutant K105R and TTP WT to similar extents, while appearing not to affect the TTP mutant S60A at all (Fig. 5A). Interestingly, PKM2 had no effect on the phosphorylation level of TTP S60A mutant (Fig. 5B) while the phosphorylation levels of wild-type TTP ascended (Fig. 4A), which suggested that the phosphorylation of TTP S60 residue was important to PKM2-mediated degradation.

TTP Ser60 is a well-studied residue that is phosphorylated by MAPK activated protein kinase 2 (MAPKAK2 or MK2). Consequently, we next wanted to confirm whether the p38 MAPK/MK2 signaling pathway participated in PKM2-mediated TTP degradation. Cells transfected with Flag-tagged TTP in the presence or absence of HA-tagged PKM2 protein were treated with the p38 inhibitor SB203580 or p38 siRNA, then harvested to detect TTP protein levels^31^. Figure 5C,D shows that, after SB203580 or p38 siRNA treatment, TTP protein levels of PKM2 overexpression groups did not differ as much as the control groups, indicating that SB203580 and p38 siRNA inhibited PKM2-mediated TTP degradation partially. These results suggest that TTP Ser60 may be an pivotal site in PKM2-induced TTP degradation and that p38 MAPK/MK2 is involved in the process.

### Manipulation of PKM2 protein levels lead to interrelated mRNA decay of TTP-associated transcripts

**Figure 6:**
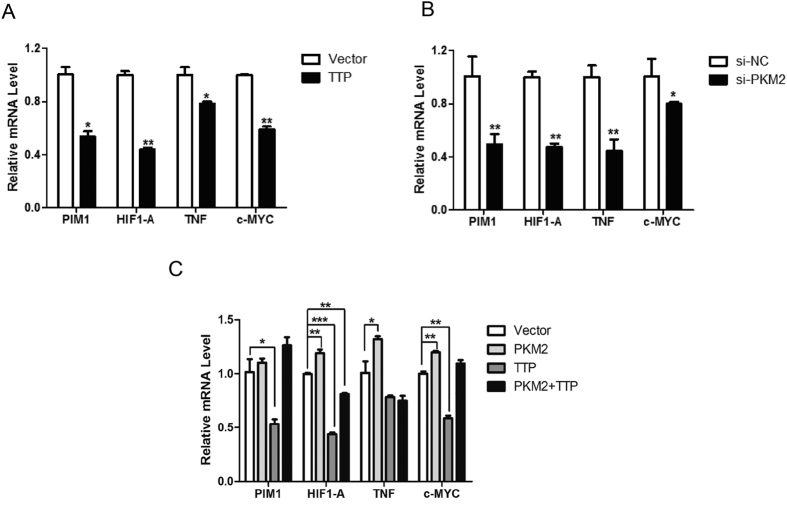
Manipulation of PKM2 protein levels lead to interrelated mRNA decay of TTP-associated transcripts. (**A,B**) Cells were transfected with Flag-tagged TTP or PKM2 specific siRNA, after 48 h incubation, qRT-PCR was performed to analyze TTP-targeted mRNAs level. (**C**) Cells were transfected with HA-tagged PKM2 or Flag-tagged TTP or both. 48 h after transfection, qRT-PCR was performed to analyze TTP-targeted mRNAs level. (Data represent mean ± SEM n = 3).

Our earlier experiments revealed that PKM2–TTP association dramatically influenced TTP protein stability. Therefore, we next wanted to determine whether this association impaired TTP function. In terms of TTP-mediated regulation of mRNA stability, we initially tested whether PKM2 knockdown affected TTP-targeted mRNA turnover as TTP protein overexpression. Many TTP-associated mRNAs are associated with immunity and tumor proliferation. We selected four mRNAs that are well studied in cancer: *PIM1*, *HIF1A*, *TNF*α, and c-*MYC*^34^. We transfected cells with Flag-TTP or PKM2 siRNA; 48 h after transfection, cells were harvested to detect mRNA levels by semi-quantitative RT-PCR. As shown in Fig. 6A,B, the TTP-targeted mRNAs decreased with the PKM2 knockdown, and these results are in accordance with the TTP overexpression data. Correspondingly, PKM2 overexpression increased the levels of these mRNAs; more importantly, the TTP-mediated decrease was abolished to some degree in the presence of the PKM2 protein. These data suggest that PKM2 leads to turnover of the mRNAs which are revealed as targets of TTP.

### PKM2-related TTP degradation affects breast cancer cell viability

**Figure 7:**
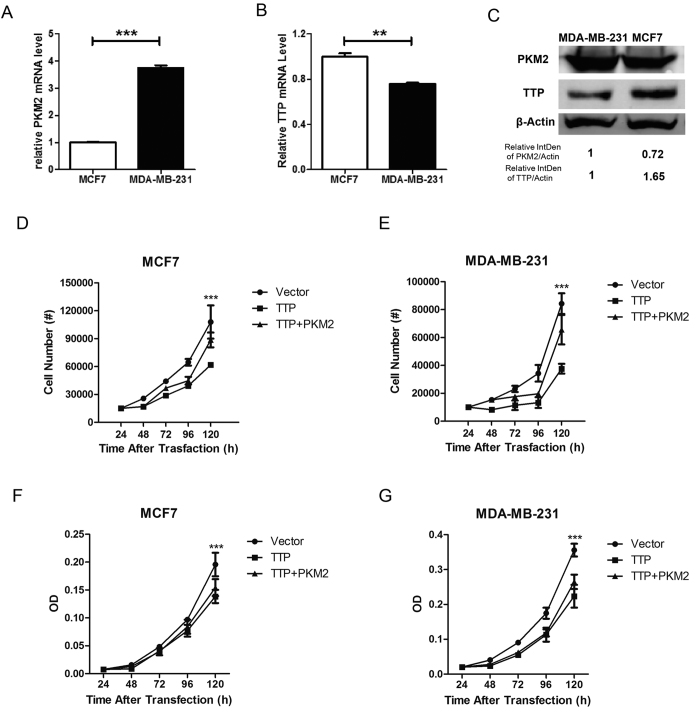
PKM2-related TTP degradation affects breast cancer cell viability. (**A,B**) MCF7 and MDA-MB-231 cells were harvested to detect the mRNA level of endogenous TTP and PKM2. (**C**) Western blotting was performed to detect the endogenous TTP and PKM2 protein level. Samples derived from the same experiment and gels were processed in parallel. The gels have been run under the same experimental conditions, and then were cut to incubate in different antibodies, full-length gels are presented in Supplementary Fig. S2. (**D,E**) MCF7 and MDA-MB-231 cells were transfected with Flag-tagged TTP or both Flag-tagged TTP and HA-tagged PKM2. 24 h after transfection, cells were replanted in 24-well plates and cell numbers were counted every 24 h for cell proliferation assays. (**F,G**) MCF7 and MDA-MB-231 cells were transfected with Flag-tagged TTP or both Flag-tagged TTP and HA-tagged PKM2. 24 h after transfection, cells were replanted in 96-well plates and treated with CCK8 buffer for 1 h then detected the OD value every 24 h. (Data represent mean ± SEM n = 3).

TTP expression in breast cancers is related to the degree of malignancy^27^. To determine whether the PKM2 expression level was relevant to TTP in breast cancer, we examined the mRNA and protein levels of endogenous TTP and PKM2. TTP mRNA and protein levels were lower in MDA-MB-231 cells as compared to MCF-7 cells, which was the diametric opposite to that of PKM2 expression (Fig. 7A–C). TTP is known as a tumor suppressor, and it affects cell proliferation in many cancers; as PKM2 impaired TTP functions, we hypothesized that PKM2 probably retarded the effect of TTP on cancer cell proliferation. As expected, overexpressing TTP significantly inhibited MCF-7 and MDA-MB-231 cell growth, while PKM2 expression diminished the TTP-mediated growth inhibition (Fig. 7D–G). Our data showed that the changes in the rate of survival of the MDA-MB-231 cell line were a little more remarkable than that of the MCF-7 cell line. A likely explanation for this was that basal TTP expression in MDA-MB-231 cells was lower than that in MCF-7 cells, which rendered MDA-MB-231 cells more sensitive to the effects of TTP.

## Discussion

PK is a key rate-limiting enzyme of glycolysis, and its isoform PKM2 plays a central role in cancer cell metabolism. Previous studies have demonstrated that PKM2 is critical for tumor growth, cell cycle progression, maintaining the malignant phenotype, and promoting cell migration^35^. In cancers, the significant multi-ability enzyme PKM2 still has functions that remain to be discovered.

In this study, we used PKM2 as the bait to screen its binding proteins in a yeast two-hybrid system, and identified TTP as a new binding partner of PKM2. The interaction between TTP and PKM2 was confirmed through independent yeast two-hybrid, co-immunoprecipitation, and GST pull-down assays, suggesting TTP directly interacts with PKM2. Next, we fragmented TTP and found that PKM2 interacted with TTP via the 51–173 aa region. Further, we found that PKM2 destabilized TTP protein and dramatically decreased exogenous and endogenous TTP. Interestingly, the changes of *TTP* mRNA levels were opposite to the protein levels. As previous studies have shown that *TTP* mRNA is a separate target of its own, we hypothesized that the PKM2 protein did not affect *TTP* mRNA directly, but TTP itself functioned as a negative feedback regulator when its protein levels decreased. Consequently, *TTP* mRNA levels increased while that of TTP protein decreased under PKM2 regulation.

Our data showed that PKM2 overexpression increased TTP phosphorylation at both serine and threonine sites. Meanwhile, TTP ubiquitination increased followed by its phosphorylation, and promoted proteasome degradation of TTP. Lee and colleagues found that TTP phosphorylation induced by a casein kinase 2 (CK2) inhibitor led to TTP degradation through the ubiquitin/proteasome pathway^31^, their work is consistent with ours but is opposite to that of others who demonstrated that TTP phosphorylation by the p38 MAPK/MK2 pathways prevents proteasome degradation of TTP protein. It is possible that the level of 14-3-3 protein expression is insufficient for protecting the phosphorylated TTP from degradation by the ubiquitin/proteasome pathway^19^,^31^,^36^. Another possible explanation for this discrepancy is that PKM2 bound to TTP and blocked the 14-3-3 proteins from associating with TTP. More detailed analysis of the role of PKM2-mediated 14-3-3 protein binding in TTP protein stability may provide insight into the relationship between TTP phosphorylation and degradation in future works.

The PKM2 kinase-deficient mutant K367M impaired TTP protein levels as much as wild type PKM2, which indicated that PKM2 kinase was not involved in this process. As PKM2 does not phosphorylate TTP itself, another kinase may be involved in the degradation. Interestingly, we found that PKM2 could not degrade TTP S60A mutant, and PKM2 had no effect on the phosphorylation level of TTP S60A mutant while the phosphorylation levels of wild-type TTP ascended. So PKM2 might have impact on phosphorylation of TTP at serine 60 residue mediated by p38 MAPK/MK2 pathway. Except for the phosphorylation level of TTP serine residues, phosphorylation level of TTP threonine residues increased, too. This result speculated that other sites may be involved in the interaction which need further investigations in our future works. Further, inhibiting p38 abolished the degradation induced by PKM2 overexpression, suggesting that the p38 MAPK/MK2 signaling pathway might participate in PKM2-induced TTP degradation. Further studies are required to unravel the detailed mechanisms involved.

As TTP is a well-known mRNA decay protein, we tested whether PKM2 association impaired this function as well. The TTP-targeted mRNAs decreased with PKM2 manipulation. More importantly, TTP-mediated mRNA decay was decreased to some degree in the presence of PKM2. We also found that, in two breast cancer cell lines with different degrees of malignancy, TTP mRNA and protein expression was lower in invasive breast cancer MDA-MB-231 cells comparing with MCF-7 cells, which was opposite to PKM2. Under PKM2 and TTP protein levels manipulation, MDA-MB-231 cells were more sensitive to TTP/PKM2 regulation than MCF-7 cells, suggesting this might be because the lower TTP expression in the MDA-MB-231 cells induced a more pronounced response to TTP overexpression^37^,^38^.

All our results indicate that PKM2 interacts with TTP directly, regulates TTP transcriptional modification, destabilizes TTP, and then impairs cell proliferation in breast cancer. Collectively, our results offer evidence of a role for PKM2 in TTP regulation in cancer cells which may be a potential therapy target in breast cancer.

## Materials and Methods

### Yeast Two-Hybrid Screening

The full-length, one N-terminal (1–354 aa) and two C-terminal portions (354–531 aa, 406–531 aa) of human PKM2 were cloned into yeast expression vector pGBKT7 (Clontech). The vectors were used as baits for the screening of Human Kidney cDNA Library (Catalog No. 638816, Clontech). The screening for the interacting protein candidates by yeast two-hybrid was performed according to the manufacturer’s instructions (Clontech)^4^,^28^,^39^.

### Cell Culture and Transfection

All cell lines including HEK293T, MCF7 and MDA-MB-231 were cultured in DMEM (GIBCO) supplemented with 10% FBS (GIBCO) at 37 °C in humidified atmosphere of 5% CO_2_. Transfection of plasmids or siRNAs were performed by Lipofectamine 2000 (Invitrogen) following the manufacture’s instruction. Full-length PKM2, kinase-deficient PKM2(K367M) were both cloned into pCDNA3/HA and full-length TTP, mutant TTP(S60A) and TTP(K105R) were cloned into pCDNA3/Flag (Vazyme). PKM2 siRNA was purchased from Genepharma generated with 5-CATCTACCACTTGCAATTA-3 oligonucleotide targeting exon 10 of the PKM2 transcript^5^ and p38 siRNA was generated with 5-CAGTCCATCATTCATGCGAAA-3 oligonucleotide^40^.

### GST pull-down assay

The GST alone, GST-tagged and His-tagged proteins were purified from E.coli BL21 (DE3) system. The GST-tagged proteins were enriched by Glutathione-Sepharose 4B beads (Amersham Biosciences) according to the manufacturer’s instructions (Amersham Biosciences). His-tagged proteins were prepared and purified using Ni-affinity resins (Merk). His-TTP protein was mixed with GST or GST-PKM2 fusion proteins in PBS binding buffer (Takara’s PBS, pH 7.4) at 4 °C for 2 h, followed by the additional 20 ml of Glutathione-Sepharose 4B beads. After 1 h incubation, beads were washed by PBST five times. Proteins pulled-down were detected by western blots as previously described^41^.

### Immunoprecipitates and Antibodies

For coimmunoprecipitation experiments, HEK293T cells were lysed by IP cell lysis buffer (Beyotime) containing certain protease inhibitors. Whole cell lysate were incubated with indicated antibodies together with 20 ul protein A plus-agarose (Pierce) overnight at 4 °C. Immunoprecipitates were washed five times and resuspended in 20 ul of 2X SDS loading buffer, then resolved by SDS-PAGE after heated at 100 °C for 10 min. Antibodies and IgG used in this study were purchased as follows: anti-TTP (Sigma), anti-PKM2 (Abcam), anti-HA (Abmart), anti-Flag (Abmart and GeneTex), anti-β-actin (Sigma), anti-phospho-Serine (Invitrogene), anti-phospho-Threonine (Cell Signaling), Goat anti-Mouse second antibody IRDye 800CW (LI-COR) and Goat anti-Rabbit second antibody IRDye 680RD (LI-COR), normal Mouse IgG (Santa Cruz).

### Detection of Phosphorylation

HEK293T cells were transfected with pcDNA3/Flag-TTP with or without pcDNA3/HA-PKM2. At 48 h post-transfection, cells were harvested and lysed with IP cell lysis buffer (Beyotime) containing certain protease inhibitors. Proteins in the cell lysates were immunoprecipitated with Anti-Flag affinity gel (Sigma) overnight at 4 °C. Proteins in the immunoprecipitates were resolved using SDS-PAGE, followed by western blotting with anti- serine or threonine phosphorylation antibody.

### Cycloheximide-based Protein Chase Experiment

HEK293T cells were transfected with pcDNA3/Flag-TTP with empty vector or pcDNA3/HA-PKM2. At 36 h post-transfection, cells were incubated with 100 ug/ml cycloheximide (CHX) (Sigma) to stop protein synthesis. Cells were harvested at 0, 1, 3, 5, and 10 h after addition of CHX, and total cell lysates were analyzed using immunoblotting with anti-Flag antibody.

### Detection of Ubiquitinylation

HEK293T cells were co-transfected with pcDNA3/Flag-TTP and pcDNA3/HA-Ub with or without HA-PKM2. At 36 h post-transfection, cells were treated with 20 ug/ml MG-132 (Selleck) and lysed with IP cell lysis buffer (Beyotime) containing certain protease inhibitors. Proteins in the cell lysates were immunoprecipitated with Anti-Flag affinity gel (Sigma) overnight at 4 °C. Then the immunoprecipitates were resolved using SDS-PAGE, followed by western blotting with anti-HA antibody.

### RNA Extraction and Semi-Quantitative RT-PCR

Total RNA was isolated by TRIZOL kit (Omega), and cDNA was synthesized by the cDNA synthesis kit (Takara). Quantitative real-time PCR was performed using the SYBR Green PCR Master Mix (Takara) on the Roche 480 system (Roche). Data were normalized to expression of a control gene (β-actin) for each experiment. PKM2 and TTP mRNA concentrations of MCF7 and MDA-MB-231 cells were calculated by comparison of threshold cycle numbers (Ct) to standard curves and normalized to endogenous β-actin mRNA levels. Primer sequences used in qRT-PCR assays in this research are listed in Table S1.

### Cell Proliferation Analysis

MCF7 or MDA-MB-231 cells were transfected with pcDNA3/Flag-TTP or pcDNA3/HA-PKM2 or both. After 24 h incubation, 1 × 10^4^ cells were harvested and reseeded onto 24-well plates, and cell numbers were counted every 24 h over a four-day period.

### Statistical Analysis

The experiments were done at least three times. And error bars were represented mean ± SEM for triplicate experiments. We determined the significance of differences in this research using Pearson’s correlation test, Student’s t test (two-tailed) or two-way ANOVA by Graphpad Prism 5. P value less than 0.05 was considered significant. Statistical significance is displayed as *P < 0.1, **P < 0.05, ***P < 0.01.

## Additional Information

**How to cite this article**: Huang, L. *et al.* Interaction with Pyruvate Kinase M2 Destabilizes Tristetraprolin by Proteasome Degradation and Regulates Cell Proliferation in Breast Cancer. *Sci. Rep.* **6**, 22449; doi: 10.1038/srep22449 (2016).

## Supplementary Material

Supplementary Information
