# Supplementary material for: Interaction with Pyruvate Kinase M2 Destabilizes Tristetraprolin by Proteasome Degradation and Regulates Cell Proliferation in Breast Cancer
Source: Sci Rep. 2016 Mar 1;6:22449. doi: 10.1038/srep22449 (PMC4772106; doi:10.1038/srep22449)
Supplement: Supplementary Information [file srep22449-s1.pdf]

Supplementary Information

Interaction with Pyruvate Kinase M2 Destabilizes Tristetraprolin by Proteasome Degradation and Regulates Cell Proliferation in Breast Cancer

Liangqian Huang[1,2] , Zhenhai Yu[2,3], Zhenchao Zhang[1] , Wenjing Ma[1,2], Shaoli Song[2] , Gang Huang[1,2*]

[1]Institute of Health Sciences, Shanghai Institutes for Biological Sciences (SIBS), Chinese Academy of Sciences (CAS) & Shanghai Jiao Tong University School of Medicine (SJTUSM), Shanghai 200025, China.

[2]Department of Nuclear Medicine, Renji Hospital, School of Medicine, Shanghai Jiao Tong University, Shanghai 200127, China.

[3]School of Biomedical Engineering, Shanghai Jiao Tong University, Shanghai 200030, China.

*Correspondence to huang2802@163.com

Table S1.Primer sequences used in qRT-PCR assays

Name	Sequences
**PKM2**	Fw: 5'-GTCGAAGCCCCATAGTGAAG-3’
	Rev: 5’-GTGAATCAATGTCCAGGCGG-3’
**TTP**	Fw: 5’-CCATCCGACCATGGAGGGACTGAGT-3’
	Rev: 5’-TTGCAGTGGGCGAAGTGGGTGA-3’
**PIM1**	Fw: 5’-CAGCAGCAGCAGCAGCAACCACTA-3’
	Rev: 5’-TTGGTGGCGTGCAGGTCGTT-3’
**TNF**	Fw: 5’-AGACGCTCCCTCAGCAAGGA-3’
	Rev: 5’-TCCCGGATCATGCTTTCAGT-3’
**HIF-1α**	Fw: 5’ CATGTGACCATGAGGAAATG 3’
	Rev: 5’ GTTGGTTACTGTTGGTATCATA 3’
**c-Myc**	Fw: 5’-GAAGGGCAGGGCTTCTCAGAGGCTT-3’
	Rev: 5’-TATTCGCTCCGGATCTCCCTTCCC-3’
**β-actin**	Fw: 5'-ACCAGGACAGCCAATACAAG-3’
	Rev: 5'-CCTCGGTCACTCATCTTCAC- 3’

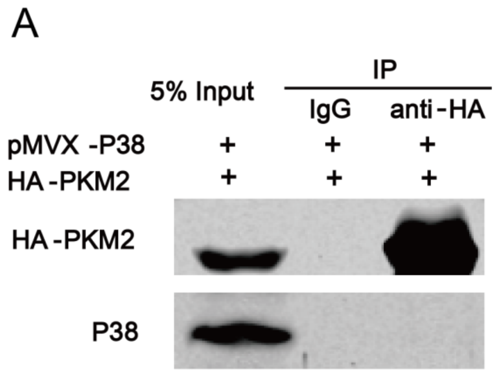

Figure S1 A. Interaction between p38 and HA-tagged PKM2 full-length proteins was examined by Co-IP followed by western blotting using anti-HA antibody or anti-p38 antibody. PKM2 and p38 proteins were over-expressed in HEK293T cells by transient transfection.

Full unedited gels for Figures 1, 2 and 3

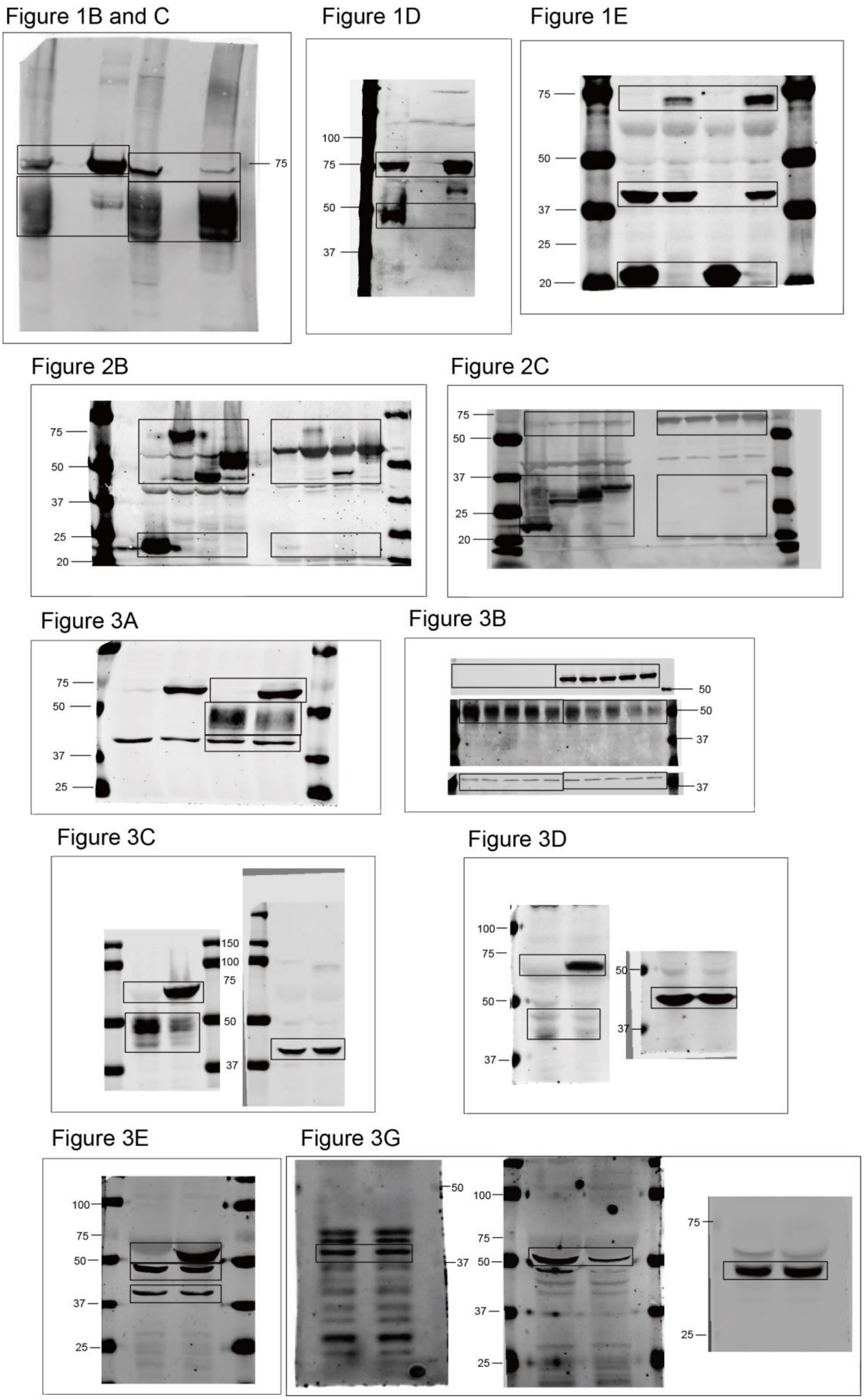

Figure S2 Uncropped Western blots for all the immunoblotting data presented in main figures and supplementary figures are shown. Molecular size markers (kDa) are indicated on the side for each blot.

Full unedited gels for Figures 4, 5 ,7 and S1

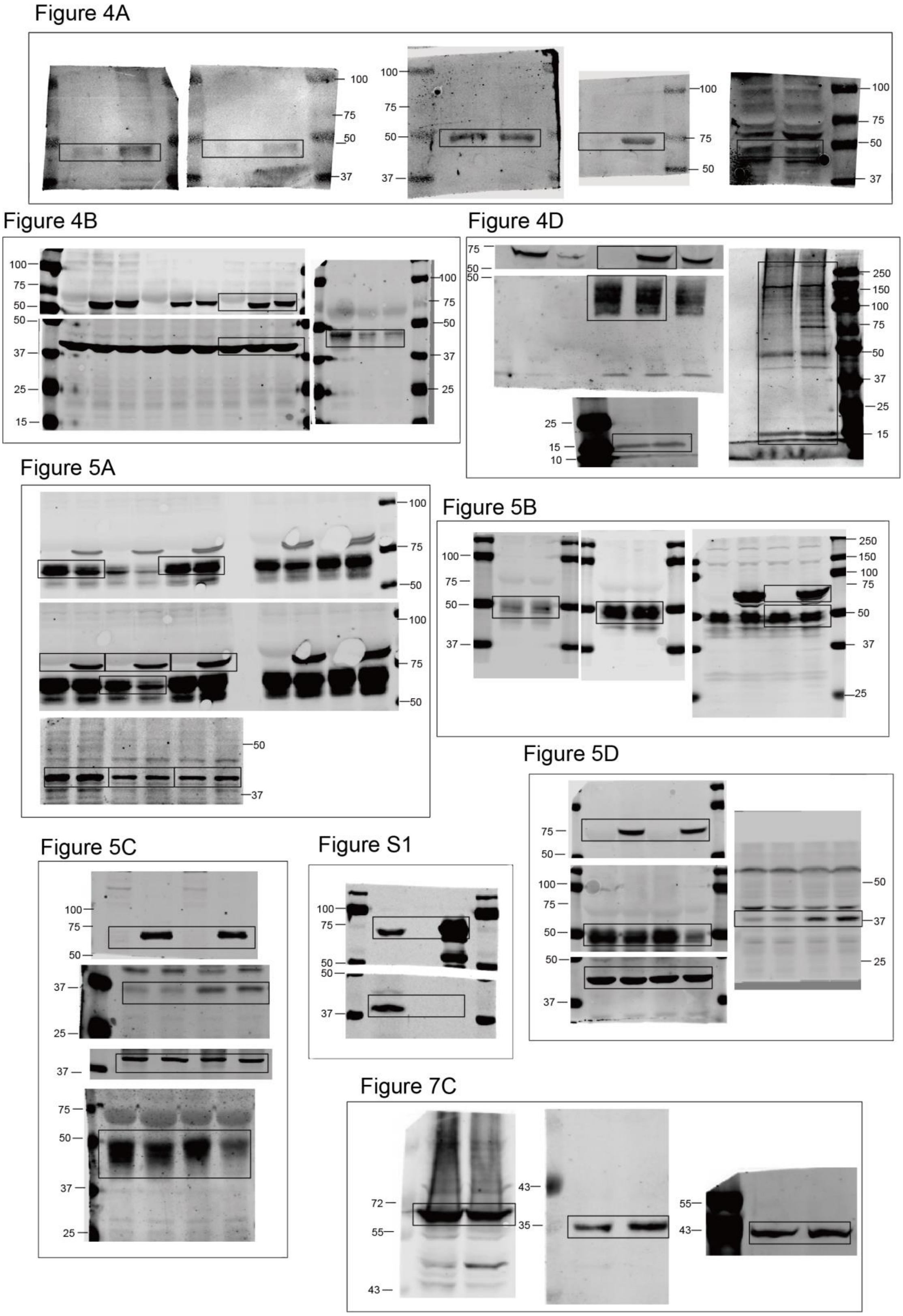

Figure S2 continued
